# A Fairness Oriented Neighbor-Channel-Aware MAC Protocol for Airborne Sensor Networks

**DOI:** 10.3390/s17051130

**Published:** 2017-05-16

**Authors:** Xiaolin Gao, Jian Yan, Jianhua Lu

**Affiliations:** 1 Department of Electronic Engineering, Tsinghua University, Beijing 100084, China; gaoxl11@mails.tsinghua.edu.cn (X.G.); lhh-dee@tsinghua.edu.cn (J.L.); 2 Beijing Aerospace Control Center, Beijing 100094, China; 3 Tsinghua Space Center, Tsinghua University, Beijing 100084, China

**Keywords:** airborne sensor networks, media access control, fairness, neighbor-channel-aware, back-off

## Abstract

In airborne sensor networks (ASNs), the media access control (MAC) protocol faces a serious unfairness problem due to the traditional protection mechanism of air-to-air communications among aircraft. Actually, by using the binary exponential back-off algorithm at high traffic loads to minimize collisions among users, the latest successful node can always benefit from this kind of MAC to obtain channel resources. Moreover, when taking the existence of the hidden nodes in ASNs into account, the inaccurate traffic load information will further aggravate the system’s unfairness. In this paper, a neighbor-channel-aware (NCA) protocol is proposed to improve the fairness of MAC protocol in ASNs. In the proposal, the NCA frame is firstly added and exchanged between neighbor nodes periodically, which helps to resolve the inaccurate traffic load information, so as to avoid reducing the probability of successful message transmission. Then a traffic-loading based back-off algorithm is involved to make the neighbor nodes cooperatively adjust the inter-frame space (IFS) interval to further reduce the unfairness. The simulation results show that, the proposed MAC protocol can guarantee the satisfied fairness, simultaneously avoiding heavy network overloads to protect key messages’ successful transmissions in ASNs.

## 1. Introduction

With the in depth research on wireless sensor network (WSN) applications, in recent years the application scene has gradually extended to the three-dimensional scene, and produced some new research areas, such as airborne sensor networks for air pollution monitoring [1] or wildfire detection and monitoring [2], underwater sensor networks for marine environment monitoring [3] etc. In airborne sensor networks (ASNs), for several decades now information has been shared among manned and unmanned aircraft, as well as surface and ground platforms, to provide protected air-to-air and air-to-ground communications for civil services [4]. Different from traditional WSNs, ASNs have several unique features, including large coverage areas, highly dynamic network topologies, unstable communication channels in the air, etc. One of the biggest challenges of designing ASNs is how to ensure a large number of geologically distributed nodes can simultaneously and efficiently access the shared communication channel with some notion of fairness among aircraft. With the fairness gradually becoming one of the important performance indicators for the network system, its related research has become a hot spot. In [5], a token-based media access control (MAC) protocol as well as a time division multiple access-based (TDMA-based) protocol called Token-MAC for passive radio frequency identification (RFID) systems was proposed to solve the unfairness and low throughput of the current contention-based MAC protocols. Reference [6] evaluated the performance of several secondary network (SN) coexistence schemes in terms of fairness among the coexisting SNs. The proposed coexistence scheme could achieve throughput and energy efficiency gains, while maintaining fairness among the coexisting SNs. In Reference [7], the authors discussed the present uplink limitations due to the inherent fairness design of IEEE 802.11 distributed coordination function (DCF) by employing the carrier sense multiple access with collision avoidance (CSMA/CA) scheme with a binary exponential back-off (BEB) algorithm, and proposed the solution of offloading the uplink traffic using IP Flow Mobility (IFOM). The proposed uplink access scheme for IFOM combined weighted proportional fairness in the Wi-Fi access and price-based resource allocation in the long term evolution (LTE) upload, which improved the energy efficiency of the user equipments (UEs) and increased the offloaded data volume under the concurrent use of access technologies that IFOM allows.

Moreover, due to the strict demands of timeliness and reliability of the time-sensitive information, and the existence of propagation delay in ASNs, the MAC protocol for ASNs must be optimized to meet the requirements of low latency and the end-to-end success ratio of the time-sensitive information. In previous studies on MAC protocols for WSNs, there were two categories: the scheduled MAC protocols and the random access protocols. In the scheduled MAC protocols, a node is explicitly assigned transmission time slots to ensure its Quality of Service (QoS), which can precisely control active nodes or links for transmission at any time by scheduling information [8,9]. Therefore, with the scheduled MAC each active link can obtain higher throughput. On the contrary, in the random access protocols, the WSNs can enable lower latency operations with less node coordination [10]. However, due to the lack of precise coordination, the random access MAC suffers from excessive collisions, and accordingly a kind of adaptive random access MAC protocols for traffic loads in ASNs is proposed [11,12,13,14]. The basic idea of adaptive random access MAC is to collect traffic loads in channel and wait for a random time by employing the binary exponential back-off (BEB) algorithm at high traffic loads, which helps to reduce the traffic load and minimize collisions while maintaining system stability. The BEB algorithm always favors the latest successful nodes, while keeping for other nodes under starvation, which obviously results in the short-term unfairness of the MAC [15]. Furthermore, the hidden nodes would aggravate the above unfairness [15,16]. Specially, in [14] a receiver-based time constraint model is built to help calculate precise network loadings to adaptively adjust the access threshold, but the fairness among all nodes cannot be guaranteed. In order to improve fairness among all nodes, the size of contention window of MAC layer is adjusted by collision statistics [17,18,19,20,21] or handshake mechanisms [22,23,24,25]. Specifically, by accurately adjusting the inter-frame space (IFS) interval in the MAC layer, a better fairness performance could be achieved [21]. Unfortunately, due to the significantly increased propagation delay caused by retransmissions under collisions, the above modified MAC cannot be directly applied in ASNs.

In this paper, a neighbor-channel-aware (NCA) MAC for ASNs is proposed to improve the MAC fairness with low overhead. The highlights of the proposed protocol lie in two aspects. Firstly, by adding NCA frame and exchanging NCA between neighbor nodes periodically, all nodes could receive their neighbors’ actual channel load information (CLI). Simultaneously, the problem of the inaccurate traffic load caused by the hidden nodes also can be solved. Secondly, a novel back-off algorithm facilitates the neighbor nodes to cooperatively adjust the MAC layer IFS interval based on the traffic load. Thus the unfairness problem caused by the BEB algorithm and the hidden nodes is tentatively resolved. Furthermore, by avoiding network overloading, the end-to-end success ratio of the packets transmission among active nodes can be sufficiently improved, with decreased delays in the process of the retransmission. In our proposal, the added NCA frames and exchanging operations show an asymptotically decreasing property under a normalized protocol overhead, which is quite different from the collision statistics and handshake which are effective.

In the following parts of this paper, the network model and the unfairness problem in ASNs will be firstly presented in Section 2, based on which a new MAC protocol will be proposed in detail in Section 3. Then comes the simulation results in Section 4. Finally, conclusions are given in the last section.

## 2. The Unfairness Problem in ASNs

### 2.1. The Network Model

**Figure 1:**
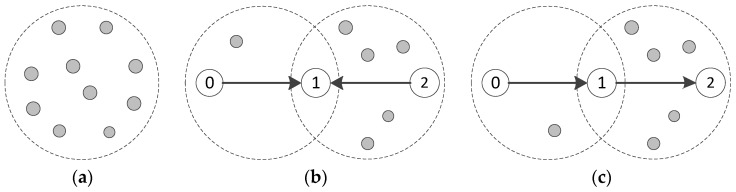
Three topology scenarios: (**a**) High contention topology, (**b**) The hidden nodes topology, (**c**) The string topology.

In this paper, we consider an aeronautical ad hoc network model as described in [11], where frequency/time-hopping technology techniques are used in the physical layer to reduce the effects of interference and increase the overall throughput and a MAC protocol adaptive to traffic loads is applied to provide the QoS guarantee. The network topology is shown in Figure 1a.

**Figure 2:**
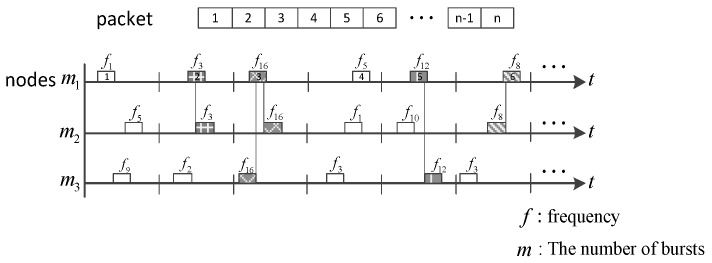
Illustration of burst collision.

All nodes in this topology scenario have the same function and are assumed to be randomly distributed geometrically. In each node, two kinds of packets are generated: The small number of low-latency packets and the larger number of normal-latency packets. Then packets are pushed into two different queues and adhere to the First-In-First-Out (FIFO) principle. The packets are split into several bursts before being transmitted, and the transmission time and frequency (Time-Hopping and Frequency-Hopping) for each burst are randomly chosen. At the receiving terminal, when bursts coming from different transmission nodes overlap in terms of time and frequency, burst collision is thought to take place. Figure 2 is the schematic diagram of the collision among three packets when they are transmitted into the channel simultaneously. Though a portion of the bursts collide, by channel coding technology, burst technology and asynchronous frequency hopping, packets would still be received correctly if the number of colliding bursts is less than the error correcting capability of the channel coding.

In order to increase the success ratio of the packets transmission in such an ASN, nodes would set the access threshold to maintain the channel traffic loads in a stable range. On the one hand, the low-latency packets should be transmitted immediately. On the other hand, before transmitting the normal-latency packets, the nodes collect bursts of channel to calculate the traffic loads and compare it with the setting access threshold. If the value of traffic loads is lower than the access threshold, which means the current channel is not overloaded, then the normal-latency packet is transmitted immediately. Otherwise, the back-off mechanism is launched. In our proposal in this paper, we still use the receiver-based time constraint model to build up a mapping relation between channel traffic loads and packet transmission success ratio, the calculation method for the access threshold is as described in [9]. It should be noted that the topology shown in Figure 1a is an ideal topology, in which all nodes are within each other’s transmission range. Actually, the ASNs are typically multi-hop networks. In this paper, the topology with the hidden nodes and the string topology scenarios (shown in Figure 1b,c, respectively) are also considered. In the dashed circle, the nodes are in the transmission range of each other, and are called neighbor nodes. Obviously, the traffic loads in each node’s channel is different due to the different numbers of neighbor nodes. Due to the high contention in high offered loads condition, the nodes have to adopt the back-off algorithm to adjust the IFS interval to retry accessing channels.

Therefore, in the back-off phase, the node adopts the BEB algorithm to compute a random back-off time to retry an access channel, which is defined as the overload avoidance process. In general, in the overload avoidance process the back-off counter is chosen in the range (0, cw−1) randomly. Thus, cw is defined as the contention window sizes and it depends on the number of overload detection of the networks. At the initial phase, cw is equal to $cw_{\min}$, which is defined as the initial contention window. Then as the overload avoidance process is performed once, cw is doubled until it reaches the maximum value $cw_{\max}$, which is $cw=2^{n}cw_{\min}$. The packet will be dropped after n (the number of retry) times of access failures. The node will access a certain channel and transmit the packet when the back-off time counter reaches zero and the channel is not overloaded.

### 2.2. The Unfairness Problem

**Figure 3:**
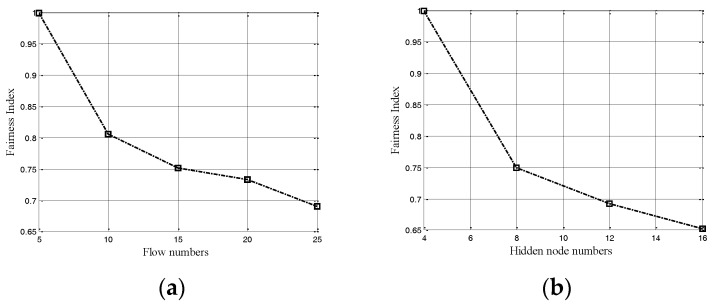

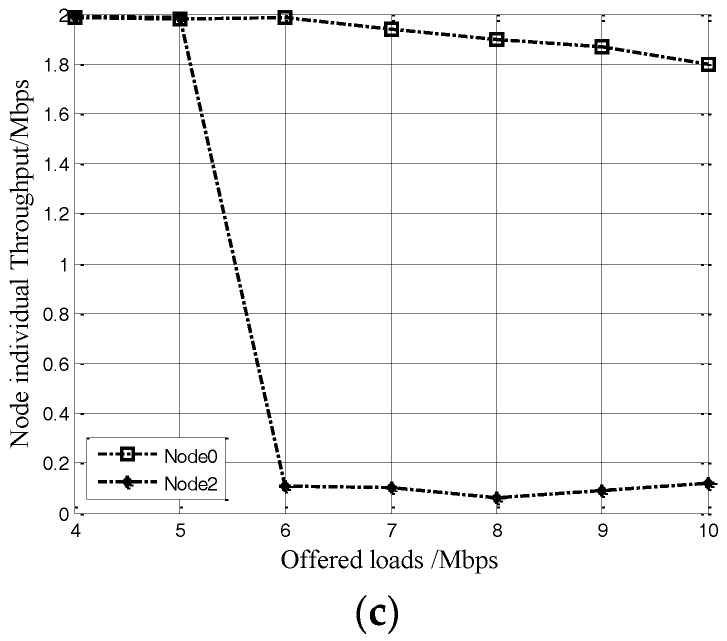
(**a**) Fairness index of high contention topology, (**b**) Fairness index of hidden nodes topology, (**c**) Starvation phenomenon in string topology.

We show the unfairness problem in this subsection. An actually fair MAC protocol in an ASN should have the following properties: (1) When the loads offered by the nodes are much lower than the system capacity, which is defined as the access threshold in this paper, the transmission request from each node should be met; (2) When nodes’ offered loads exceed the system capacity, each node would be able to get its fair share of the channels, i.e., the same throughput of each node in the condition that the loads are equally offered. That is, the fairness of the MAC protocol enables using shared media resources of the node evenly. In this paper, we use the fairness index (*FI*) defined in [26] given by:

(1) $$FI=\frac{{\left(\sum_{i}f_{i}\right)}^{2}}{n\left(\sum_{i}f_{i}^{2}\right)}$$

where  fi denotes the throughput of the flow *i*, and *n* is the total number of the flows. As the above definition of fairness, absolute fairness is achieved when *FI* = 1, while absolute unfairness is achieved when *FI* = 1/*n*. Also as in [19], the index has been averaged over all back-off time of the frames, which will be considered in our simulations. In order to show the fairness effects of different MAC protocols in ASNs, we will give here some initial numerical simulation results in the scenario shown in Figure 1. The results are presented in Figure 3, in which the fairness indexes are shown to decrease with the increase of flow numbers.

Firstly, the node topology as Figure 1a is considered, with the results shown in Figure 3a. In fact, when the offered loads of the nodes in the dashed circle (in Figure 1a) reach a higher level, the nodes have to use the BEB algorithm to contend the channel resources to transmit. When nodes do not get the chance to access the channel, they would be in overload avoidance process. In this paper, $P_{tr}$ is defined as the transmission probability of nodes, where $P_{tr}=1/\left(2^{n}\cdot CW_{min}+1\right)$. $P_{tr}$ will decrease exponentially along with *n* (the number of back-offs). In the overload avoidance process, the contention window (CW) is adjusted through the BEB algorithm to resolve the overload problem. However, because of randomness, the statistical traffic loads of the nodes may reach the access threshold consecutively in a short-time. The above inaccurate evaluation of traffic loads will lead to the overload avoidance process consecutively, thus encounter severe degradation in throughput. Therefore, the BEB algorithm is ineffective in resolving the overload problem in ASNs, by which the short-term unfairness still keeps as randomness runs through.

Then, the hidden nodes topology in Figure 1b is considered. The node 0 is hidden from node 2. Node 0 and 2 would access the channel differently in different traffic loads. For instance, node 0 would access the channel constantly in the condition of lower traffic loads. Conversely, node 2 has to contend to access the channel with its large number of neighbor nodes and encounter different degradation in throughput because of the different $P_{tr}$. Even if the traffic loads of node 0 and 2 are of the same level, the $P_{tr}$ is different due to the randomness as analyzed above. When node 0 and 2 transmit to node 1 simultaneously, the success ratio of the packets deceases intensely due to the amount of collisions in node1. The related results are presented in Figure 3b with different numbers of hidden nodes, which is also far from 1.

Finally, the string topology as shown in Figure 1c is also considered. As the loads increase to a certain degree, even if two dashed circles are in lower traffic loads, node 1 would suffer from starvation. As the nodes in the two dashed circles are their respective neighbor nodes, it results in the sum of statistical loads in the two dashed circles, which will always exceed the access threshold. This reflects the starvation phenomenon presented in Figure 3c. When the offered traffic load increases to a certain degree, node 0 will “capture” the channel and node 1 will suffer severe degradation in throughput. This starvation problem would further lead to congestion when node 1 is the intermediate of flow from node 0 to 2, which might reduce system stability. So the key problem in the above scenarios is that the transmitters could not receive the accurate traffic loads information in multi-hop share channel with hidden nodes. Then the transmit nodes can only access the channel blindly with local statistical loads information, due to the lack of accurate channel information recorded by the receivers.

So far, as can be seen in Figure 3, by traditional MAC protocols the FIs are always less than and far from 1. The problems (i.e., randomness, and inaccurate traffic loads) that cause unfairness in ASNs have been explicitly identified.

## 3. The Proposed MAC Protocol

In general, all nodes in WLAN use control frames by handshake mechanism to relieve the hidden nodes problem. Based on this idea, we add a neighbor-channel-aware (NCA) frame in the MAC layer (which belongs to the small number of low-latency packets and could be transmitted directly) to realize information sharing between the neighbor nodes, which could relieve the inaccurateness of the traffic loads caused by the hidden nodes. Different from the handshake mechanism in WLANs based on data driven, the NCA frame in our proposal will be sent periodically. In this section, the NCA-based MAC protocol is firstly presented in detail. As mentioned before, the unfairness frequently happens in overload avoidance process, so we are going to explain how to ensure the networks overload avoidance by using the NCA frame in the following subsection.

### 3.1 Networks Overload Avoidance

**Figure 4:**
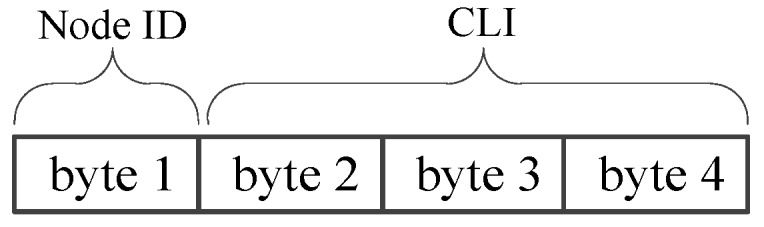
The NCA frame structure.

The NCA-based MAC frame contains the node ID and its local channel traffic loads information (CLI). The CLI is defined as the number of bursts which received by the nodes from the frequency hopping channel in a cycle. The whole NCA frame structure is shown in Figure 4, for instance, occupying 4 bytes in this paper.

The neighbor nodes exchange the NCA frames with each other periodically. When a node receives the NCA frames from its neighbor nodes, it will read the *CLI* of the NCA frames and reset its *CLI* which is equal to the maximum value of the *CLIs* (its own *CLI* and its neighbor’s *CLI*) to avoid the inaccurate *CLI* caused by the hidden nodes problem. When the node’s local *CLI* is higher than the *Max_NL* (i.e., the access threshold), it would launch the BEB back-off mechanism to avoid networks overload. However, the randomness would still lead to the unfairness of the obtained channel resources. In this paper, we further define *A_Threshold* as a new access threshold:

(2) A_Threshold=α·Max_NL

in which  α is defined as the percentage of the *Max_NL*.

The MAC protocol in ASNs used *Max_NL* as the only criterion for back-off, resulting in the unfairness of the random BEB algorithm. We introduce *A_Threshold*, with the aim of allowing the back-off time dynamically varying with the network loads among the interval [*A_Threshold*, *Max_NL*]. In such a case, the unfairness of the BEB algorithm can be meliorated by adjusting the value of *α*.

Each node checks if the *CLI* exceeds the new access threshold before the transmission. If the *CLI* exceeds the *A_Threshold*, which means that there would potentially be overload condition, then the node would have to enable the back-off algorithm to increase the IFS interval as shown in the following sub-section.

### 3.2 The Back-off Algorithm

The proposed back-off algorithm can be described as follows:

The node collects the traffic loads and compares the *CLI* to the *A_Threshold* before transmission.

Case I: If *CLI < A_Threshold*, the node would not use a back-off algorithm.

Case II: If *CLI > A_Threshold*, the node would use a back-off algorithm by counting an accurate IFS interval. The transmission of the frame would not begin until the back-off counter equals zero.

Next, we define some parameters as follows:

*R* is defined as the average number of bursts per second sent by the nodes.

*R*_min_ is defined as the minimum of *R*, and *R*_max_ was defined as the maximum of *R*.

ρ is defined as the exponential decrease of the *R*, and *R* changes in the rang [*R*_min_, *R*_max_] with the CLI changing in the rang [*A_Threshold*, *Max_NL*],

So,  ρ can be given by:

(3) $$\rho =\frac{lnR_{min}-lnR_{max}}{A\_Threshold-Max\_NL}$$

and:

(4) $$R=R_{max}\cdot e^{\rho \left(A\_Threshold-CLI\right)}$$

Thus *R* would decrease with the increase of *CLI*, which means the interval for sending two consecutive packets would increase.

$T_{IFS}$ is defined as the interval for sending two consecutive packets (also be known as the IFS interval), given by:

(5) $$T_{IFS}=\{\begin{array}{c} 0,\;CLI\leq A\_Threshold\;\\ T_{f}(\frac{1}{R}-\frac{1}{R_{max}}),\;CLI>A\_Threshold \end{array}$$

$T_{f}$ is defined as the transmission time of a frame, which is:

(6) $$T_{f}=L_{f}/R^{\prime }$$

$T_{f}$ is defined as the length of the frame in bits, and $\;R^{\prime }$ is defined as the transmit rate (bit/s), so by controlling the $T_{IFS}$, node could control the average number of transmission bursts per second.

**Figure 5:**
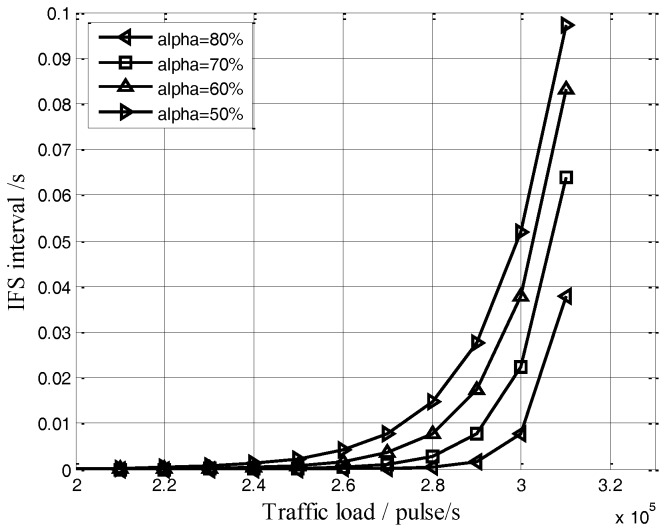
IFS interval vs. the traffic load.

Figure 5 shows the exponential increase of the IFS interval along with the traffic load increase, under the back-off parameters shown in Table 1. The IFS interval (the time of waiting to access channel) would be longer with the larger value of *α* in the same traffic loads condition.

The nodes will access the frequency-hopping channels distributively by the above real-time back-off algorithm, thereby avoiding the short-time unfairness of the BEB algorithm caused by the randomness.

### 3.3. Analysis of NCA’s Overhead and Fairness

#### 3.3.1 Protocol Overhead Analysis

**Figure 6:**
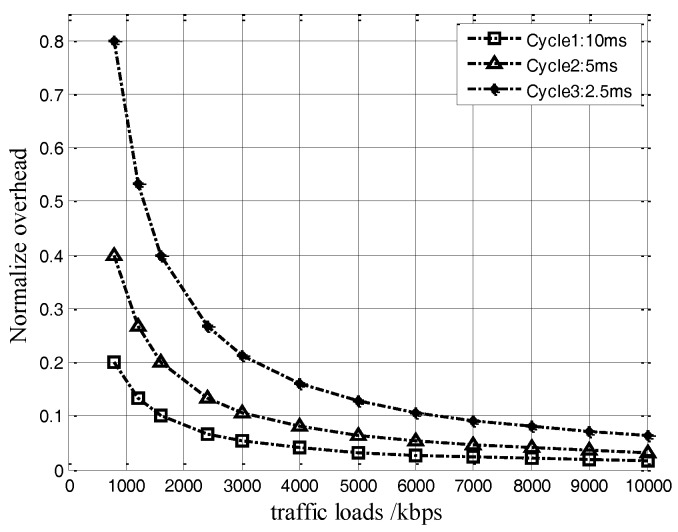
Normalized overhead of NCA-based MAC.

In our proposal, the use of NCA frames obviously indicates extra overhead increases in the original MAC protocol. Since the NCA frame is only periodically sent among neighbor nodes, the overhead size can be decided by the node number and the periodic time (cycle) of the NCA frame, but without relation to the traffic load. In theory, the protocol overhead is proportional to the node number and the periodic time of the NCA frame, i.e., $Overhead_{NCA}\propto (N_{node},T_{cycle})$. The functional relationship between the normalized protocol overhead (overhead/traffic load) and the traffic load can be shown in Figure 6 by numerical results.

In Figure 6, it can be seen that, under the same traffic load conditions, the greater the cycle, the less normalized overhead. When the traffic load increases, the corresponding normalized overhead decreases. Take the 10 ms cycle as an example, as the traffic load is increased to 2 Mbps, the normalized protocol overhead will be reduced less than 9%, which would even drop further along with the increase of traffic load. Therefore, we can conclude that, by the NCA-based MAC, the protocol overhead shows asymptotically decreasing property in ASNs.

#### 3.3.2. Fairness Analysis

We can also analyze the fairness provided by the NCA-based MAC here. Define the channel utilization of the nodes as $\frac{T_{frame}}{T_{frame}+T_{IFS}}$, where $T_{frame}$ is the actual sending time. According to the NCA frame for the accurate channel traffic load information, the node will calculate the precise IFS by $T_{IFS}$. By the calculation process in Section 3.2, each node could obtain the same $T_{IFS}$ in the same traffic load. When the frames are in the same length, the channel utilization of each node is equal, which means that the fairness of the protocol enables using shared media resources among all the nodes uniformly.

## 4. Simulations

In this section, we are going to present the simulation experiments by using opnet14.5 network simulation software in which the seed parameter in each simulation scenario is set to 1000 (that is, the number of samples is 1000), and we compare the protocol performance of the NCA-based and the traditional BEB-based MAC schemes. The receiving range of the nodes is set as its transmission range. The physical parameter is based on those of the frequency-hopping channel simulations in [14] throughout this section, which are shown in Table 2. The NCA-based back-off parameters are set as Table 1 in Section 3. The cycle time is set as 10 ms. The BEB algorithm parameters refer to the IEEE 802.11 DCF [19,20]. The CW_min_ is chosen as 15 and it has been retried for 5 times.

**Figure 7:**
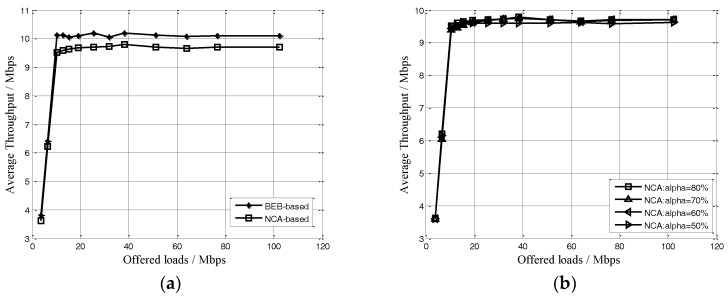
(**a**) Average throughput of two protocols, (**b**) Average throughput of NCA-bases with different α.

Before discussing the fairness performance of the protocol, we present the simulation experiments to compare the average throughput of NCA-based with different value of α and BEB-based MAC schemes. In this simulation scenario, nodes are randomly distributed in a 150 km × 150 km plane area and the number of nodes is 100. The NCA frame’s cycle equals to 10 ms, so the protocol overhead could be calculated, which equals to 0.32 Mbps. Figure 7 shows the average throughput of the NCA-based with different value of *α* and BEB-based MAC schemes.

In Figure 7a, the average throughput of the NCA-based MAC with the *α* = 80% is lower than that of the BEB-based due to the protocol overhead. Nevertheless, the difference approximately equals to the corresponding overhead, which is far less than the maximum channel loads of the system. In Figure 7b, the different of the average throughput of NCA-based with different value of α is very small, so the value of *α* would not affect throughput performance, especially in networks overload conditions. In following simulation experiments, the value of *α* is set as 80%. We still use the FI as (1) given in Section 2.2. The indicators of measuring the performance of the protocol include the FI, the average throughput and the end-to-end success ratio. To be specific, the end-to-end success ratio is defined as the total number of transmitted packets divided by the number of successfully received packets, which is one of the most important indexes in ASNs. Then we check the MAC performance under different topologies, including fully-connected topology, hidden nodes topology and string topology shown in Figure 1.

### 4.1. Fully-Connected Topology

**Figure 8:**
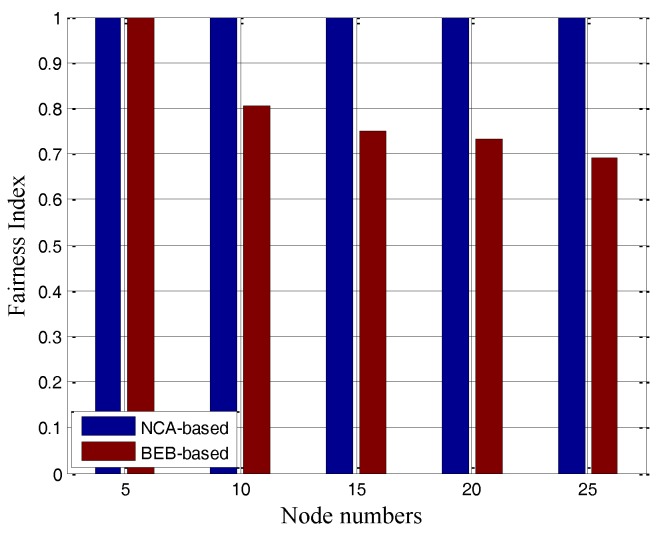
Fairness Index for the fully-connected topology.

In the dashed circle shown in Figure 1a, nodes are in the transmission range of each other. Five individual experiments with different numbers of nodes were carried out. In the simulation scenario, nodes are randomly distributed in a 150 km × 150 km plane area. For each node, the Poisson traffic is adopted and the mean rate could achieve the maximum value (2 Mbps). The unfairness occurs only when there are more than five transmissions in process simultaneously, in which the maximum networks load is 10 Mbps. The results of average aggregated throughput and end-to-end success ratio are given in Table 3, and the FIs under different MAC protocols are shown in Figure 8.

In Figure 8, the BEB-based MAC protocol results in serious unfairness problem in all cases except the first one as the channel is not filled up (i.e., when the node number is 5, all the nodes can access channels with sufficient fairness by both the BEB-based and NCA-based protocols). This is consistent with our previous analysis. In all cases of different node numbers, the FI of NCA-based MAC protocol can always approach 1 with tiny gaps, which means that most nodes in the network can share the channel resources and chances to access for successful transmissions. It makes all nodes share the wireless channel with enough fairness and stability. From Table 3, by the BEB-based and NCA-based protocols, the end-to-end success ratios can both reach up to 99%. We can also notice that the average throughput of the NCA-based MAC is a little lower than that of the BEB-based, but is still close to the maximum networks loads (10 Mbps in theory), which tells us the NCA-based MAC can guarantee the system throughput close to the ideal level.

### 4.2. Hidden Nodes Topology

We then check the performance under the hidden nodes topology as shown in Figure 1b. We set different number of nodes to generate different offered loads in the two dashed circles. For each node, the Poisson traffic is adopted and the value of its mean rate is high enough to achieve maximum rate (2 Mbps). The experiment parameters are shown in Table 4 (node amount (left) includes node 0 and node amount (right) includes node 1). Scenario 1 shows that the channel of node 0 and node 2 are both in a low traffic loads condition. Scenario 2 shows that the channel of node 0 is in a low traffic loads condition while the channel of node 2 is in a high traffic loads condition. And scenario 3 shows that both of them are in a high traffic loads condition.

**Figure 9:**
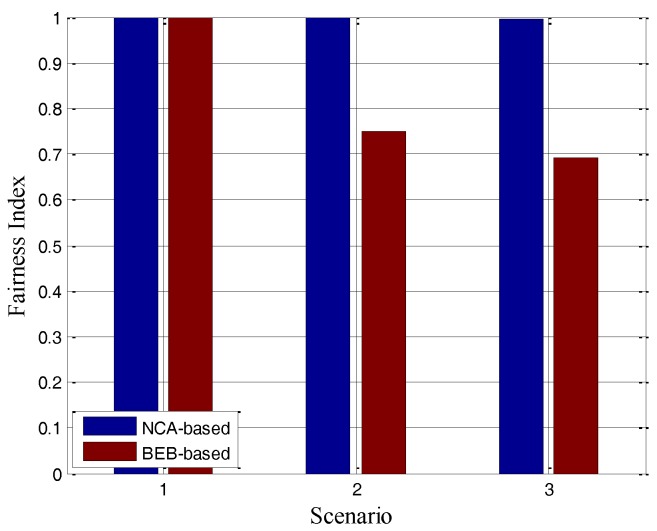
Fairness Index for the hidden node topology.

The simulation results are given in Table 5 and Figure 9. The BEB-based MAC protocol results in a serious unfairness problem and an obvious decrease of the end-to-end success ratio in scenario 2 and scenario 3. Due to the unequal traffic loads in channels, node 0 will get much higher throughput than node 1. In the contrary, the FI of NCA-based MAC protocol is close to 1 in scenarios 2 and 3, indicating that nodes would get a better share of the channel resources and more chances to access the channel to transmit even though they are hidden from each other. It makes nodes share the multi-hop wireless channels fairly and stably by receiving NCA frames and adjusting the back-off time accurately.

### 4.3. String Topology

Finally, we present the experiments of evaluation performance of the NCA-based and BEB-based schemes under the string topology, as shown in Figure 1c. In this case, one node is set in left dashed circle, and it generates Poisson traffic with the same mean rate as node 0 and 2 of 2 Mbps. Five nodes are set in the right dashed circle, which generate Poisson traffic at the same mean rate.

**Figure 10:**
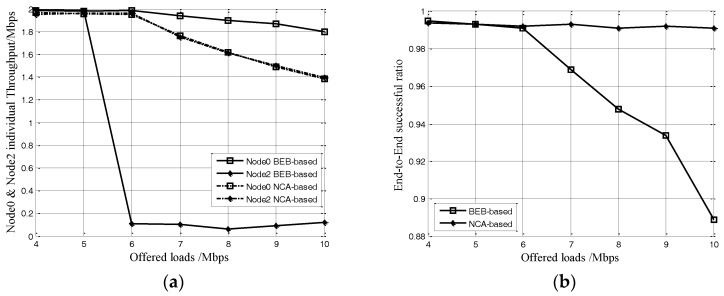
(**a**) Node individual throughput, (**b**) end-to- end success ratio for the string topology.

Figure 10 shows that the BEB-based scheme will cause a serious starvation problem for node 1 and an obvious decrease of the end-to-end success ratio when the loads offered by the five nodes in the right dashed circle reach 6 Mbps. These results are consistent with the following facts: when all nodes in the two dashed circles are node-1’s neighbor nodes, node 1 has to content with the other nodes for channel resources. As the neighbor nodes are hidden from each other, they could transmit without back-off due to the lower loads in each dashed circle, thus their transmission could fill up the node 1’s channel as the loads offered by the five nodes in the right dashed circle increase to 6 Mbps (meanwhile, the traffic load in the channel of node 1 reaches 10 Mbps). This causes node 1 to always be in the back-off phase to avoid its local channel from overloading. On the contrary, in our NCA-based scheme, if nodes in two dashed circles receive the NCA frame transmitted from node 1, its CLI will indicates its neighbor nodes with a higher proportion of channel share than what they should obtain. Its neighbor nodes would increase their back-off time accordingly. Along with the increase of nodes’ inaccurate back-off time, the node 0’s throughput can be balanced with the neighbor nodes, showing better fairness than the BEB-based one. At the same time, the end-to-end success ratio in node 1 could be maintained above 99% even under the condition of high offered loads as Figure 9b shows.

## 5. Conclusions

In this paper, the performance degradation of the MAC protocol is recognized as a compounded result of the randomness of the BEB algorithm and inaccurate traffic loads due to the hidden nodes in multi-hop ASNs. In order to solve those two problems of traditional MAC protocols of ASNs, a proposal of exchanging CLI periodically between neighbor nodes in the MAC layer is developed to overcome the inaccurate traffic loads caused by the hidden nodes. Then a modified back-off algorithm adaptive to traffic loads is used to calculate the accurate IFS interval, which helps to reduce the randomness of the BEB algorithm. In order to evaluate the MAC performance of the NCA-based protocol, some simulation experiments have been carried out to compare its performance with that of the BEB-based scheme. Simulation results show that the average throughput of NCA is lower than that of BEB. Nevertheless, the difference is far less than the maximum channel loads of the system. Simulation results also indicate that the NCA-based MAC protocol achieves better fairness and higher end-to-end success ratio, which is almost independent of the traffic loads. In addition, the NCA-based scheme could always achieve stable and high throughput under different topologies, while the BEB-based scheme performs poorly in terms of metrics for multi-hop topologies. Thus, the NCA-based MAC protocol provides a very stable link layer by the loss of a small acceptable amount of throughput, which is more adaptive and suitable for future applications in multi-hop ASNs.

## Figures and Tables

**Table 1:** NCA-based Back-off Parameters.

Parameters	Value
*R_max*	64,000 bursts/s
*R_min*	60 bursts/s
*Max_NL*	320,000 bursts/s
*α*	80%, 70%, 60%, 50%

**Table 2:** Physical simulation parameters.

Parameters	Value
Node transmission rate	2 Mbps/64,000 bursts/s
Packet length	1024 bits
Number of bursts per packet	32
Number of frequency	16
Error correction coding	1/3Turbo code If the number of bursts received per packet is no less than 18, it can be successfully recovered by turbo coding.
Access threshold	10 Mbps/320,000 bursts/s

**Table 3:** Average Aggregated Throughput and end-to-end success ratio for Fully-connected Topology.

Scenario	Average Throughput (Mbps)		End to End Success Ratio	
	NCA-Based	BEB-Based	NCA-Based	BEB-Based
10 nodes	9.836	10.105	0.99545	0.99461
20 nodes	9.821	10.089	0.99434	0.99121
30 nodes	9.838	10.018	0.99501	0.99190
40 nodes	9.830	10.102	0.99383	0.99201
50 nodes	9.818	10.055	0.99377	0.99097

**Table 4:** Experimental parameters for hidden nodes topology.

Scenario	Node Amount (Left)	Node Amount (Right)
1	2	2
2	2	6
3	6	6

**Table 5:** Node individual throughput and end-to-end success ratio for the hidden nodes topology.

Scenario	Node 0’s Throughput (Mbps)		Node 2’s Throughput (Mbps)		End to End Success Ratio	
	BEB-Based	NCA-Based	BEB-Based	NCA-Based	BEB-Based	NCA-Based
1	1.988	1.965	1.991	1.953	0.99787	0.99683
2	1.763	1.290	1.061	1.290	0.90563	0.99348
3	0.831	1.213	0.056	1.190	0.83294	0.99298
